# Deterministic and stochastic population-level simulations of an artificial *lac *operon genetic network

**DOI:** 10.1186/1471-2105-12-301

**Published:** 2011-07-26

**Authors:** Michail Stamatakis, Kyriacos Zygourakis

**Affiliations:** 1 Department of Chemical and Biomolecular Engineering, Rice University, Houston, TX 77005, USA

## Abstract

**Background:**

The *lac *operon genetic switch is considered as a paradigm of genetic regulation. This system has a positive feedback loop due to the LacY permease boosting its own production by the facilitated transport of inducer into the cell and the subsequent de-repression of the *lac *operon genes. Previously, we have investigated the effect of stochasticity in an artificial *lac *operon network at the single cell level by comparing corresponding deterministic and stochastic kinetic models.

**Results:**

This work focuses on the dynamics of cell populations by incorporating the above kinetic scheme into two Monte Carlo (MC) simulation frameworks. The first MC framework assumes stochastic reaction occurrence, accounts for stochastic DNA duplication, division and partitioning and tracks all daughter cells to obtain the statistics of the entire cell population. In order to better understand how stochastic effects shape cell population distributions, we develop a second framework that assumes deterministic reaction dynamics. By comparing the predictions of the two frameworks, we conclude that stochasticity can create or destroy bimodality, and may enhance phenotypic heterogeneity.

**Conclusions:**

Our results show how various sources of stochasticity act in synergy with the positive feedback architecture, thereby shaping the behavior at the cell population level. Further, the insights obtained from the present study allow us to construct simpler and less computationally intensive models that can closely approximate the dynamics of heterogeneous cell populations.

## Background

Since the introduction of the operon concept by Jacob et al. [1], the *lac *operon genetic switch has been considered as a paradigm for genetic regulation. Several experimental studies of this system over the past several decades have elucidated the underlying biomolecular interactions and a plethora of mathematical models have integrated the complex interplays of the key biochemical species in order to predict the behavior of the system [see, for example, [2]].

Most of these models, however, pertain to the single cell behavior [see, for example the models reviewed in [3]] with a limited number of studies focusing on cell populations, or taking comparative approaches. For instance, in a mini-review article, Vilar et al. [4] compared different models pertaining to single cells and cell populations, in order to illustrate the performance and limitations of different methodologies. At the single cell level Vilar et al. [4] simulated four phenomenological ordinary differential equations (ODEs) for the concentrations of permease, inducer, and β-galactosidase. A stochastic single cell model was then developed by transforming the phenomenological deterministic rates into propensities. However, the latter transformation is not unique, contrary to the case of a mechanistic model where the reaction rate expressions are derived on the basis of statistical mechanical assumptions [see for instance chapter X in ref. [5], and refs [6-8]]. Thus, the stochastic model simulated by Vilar et al. [4] is based on *ad hoc *assumptions. In addition, the Vilar et al. models do not appear to account for the dilution of the concentrations due to cellular growth [9], for cell division and for stochastic partitioning or DNA duplication effects, which are important sources of extrinsic noise [10-12].

Furthermore, van Hoek and Hogeweg [13,14] studied the effect of intrinsic and extrinsic sources of stochasticity, as well as genetic mutations and spatial heterogeneity, at the single-cell and the population level from an evolutionary perspective. Their deterministic model consists of an intracellular and an extracellular part, with the latter capturing the influxes and effluxes of glucose and lactose into and from the cells, as well as the diffusion of these species over a grid, the points of which are assumed to be vacant or occupied by single cells. The intracellular part of the model employs ten differential equations for the concentrations of mRNA, β-galactosidase, permease, lactose, allolactose, glucose, glucose-6-phosphate, cAMP, ATP, and the size of the cell. The rate expressions used in this model contain Hill functions to model saturation and cooperativity effects, and account for dilution due to cell growth. Hence, *stricto sensu *the model is not a mechanistic one, and the incorporation of stochastic effects invokes *ad hoc *assumptions rather than a formal approach based on statistical mechanics. In particular, van Hoek and Hogeweg [13] use the deterministic equation for the mRNA species, but interpret the mRNA concentration as the probability of a single mRNA molecule being present in the cell. They subsequently use this probability to infer the frequency of translational bursts.

Thus, there remain several open questions regarding the emergence of population behavior from the complex interplay between reaction dynamics and stochasticity at the single cell level. Investigating this connection and ultimately understanding cell population dynamics is significant for two reasons. First, typical biology experiments involve cell populations rather than single cells and, thus, phenotypic distributions obtained for instance from flow cytometry pertain to the cell population rather than the lifetime of a single cell. Second, there have been mathematical modeling studies for simple genetic networks, suggesting that the behavior of the single cell is very different from the behavior of the cell population [12,15,16]. It would therefore be interesting to investigate whether this is the case in the more complex *lac *operon system, or whether one can formulate average single cell models that can adequately capture the behavior of the cell population.

In a previously published article, Stamatakis and Mantzaris [17] have presented a kinetic scheme that captures the salient features of an artificial *lac *operon system, which can be constructed in the lab by introducing mutations to the wild-type system. In the present paper we will incorporate that kinetic scheme into two Monte Carlo (MC) frameworks that simulate cell population dynamics.

The first framework assumes stochastic reaction occurrence and takes into account stochastic DNA duplication, division and partitioning. McAdams and Arkin [18-20] were the first to employ the Gillespie MC algorithm [7,8] to simulate gene induction and protein synthesis at the single cell level. This approach was later extended to account for cell growth and division [21-23]. However, all these and many other studies simulated the dynamics of biochemical pathways at the single cell level. In a recent study we generalized and expanded the chemical master equation (CME) to the cell population level [10]. The resulting cell population master equation (CPME) is simulated with a Monte Carlo algorithm, and captures stochasticity in the intracellular reaction, stochastic DNA duplication, and division events, as well as in the partitioning of the content of a mother cell to the two daughters. This novel framework is applicable to cell populations since every single daughter cell is tracked, thereby making possible the calculation of any statistical property of the population.

In order to better understand how stochastic effects shape cell population distributions, we develop here a second framework that assumes deterministic reaction dynamics and stochastic DNA duplication, cell division and partitioning. Throughout this work, the single cell models are derived from the reaction network developed in ref. [17] by invoking standard assumptions based on statistical mechanics and reaction rate theory [7,8,24] rather than *ad hoc *techniques [4]. We also explicitly take into account the dilution effect due to cell growth in the deterministic case [9]. Furthermore, stochasticity in the times of division and DNA duplication, as well stochastic partitioning effects are effects that were neglected in previous studies [4], but are explicitly taken into account in our cell population frameworks.

Comparisons of the predictions of the two frameworks reveal that stochasticity can create or destroy bimodality, and may enhance phenotypic heterogeneity by creating heavy tailed distributions, a phenomenon that has not being shown before and can be investigated experimentally. The insights provided from the new study also allow us to construct simpler and less computationally intensive models that can closely approximate the dynamics of heterogeneous cell populations. Specifically, for the case of deterministic reaction dynamics, we use the continuum model formulation which assumes that all cells in the population occupy a continuous and expanding biotic phase [9]. Under this assumption, a set of mass balances for a representative "average" cell in the population provides a lumped description of cell population dynamics. We demonstrate an excellent agreement between the continuum and the cell population models, which is encountered for the first time and contradicts previous studies. Extending these results in the stochastic case, we show that the calculation of distributions of intensive quantities (such as species concentrations) can be performed on the basis of single cell simulations, instead of computationally demanding population-level ones.

## Methods

**Figure 1:**
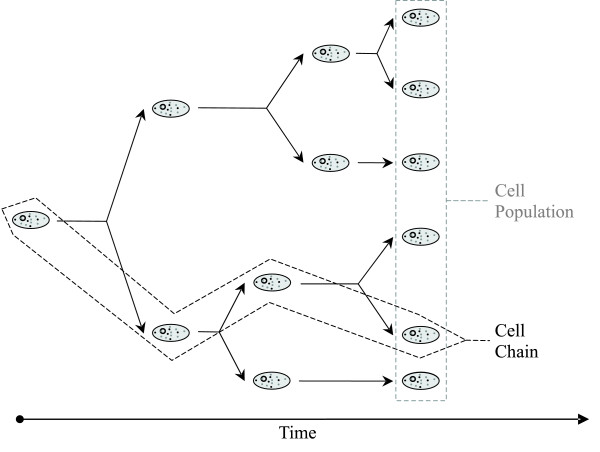
**Definition of cell chain and cell population**. A cell chain essentially stores information about the history of a single cell in time. On the other hand, a cell population consists of all the viable offspring observed at time *t*.

Figure 1 defines pictorially the concepts of "cell chain" and "cell population." A cell chain is a collection of cells defined by starting from one mother cell, choosing one of its daughter cells upon division, setting this as next mother cell and repeating the process. A Bernoulli trial with probability 1/2 governs the choice of which cell to keep after each division event. A cell chain essentially stores information about the history of a single cell in time. On the other hand, a cell population consists of all the viable offspring observed at time *t *that were generated by an arbitrary number of cells at t = 0. For simplicity, Figure 1 portrays a population that originated from a single cell. However, our definition can be applied to cases where more than one cells exist at time t = 0.

We are now ready to present the frameworks that will be used for the simulating cell populations in this study. Both frameworks treat the occurrence of division and DNA duplication events as stochastic processes. Their difference lies in the treatment of reactions and the partitioning of species between the daughter cells: the first framework treats these as stochastic, whereas the second one as deterministic processes. We will refer to the former as the "population model with stochastic reaction dynamics", and the latter as the "population model with deterministic reaction dynamics".

### Population Model with Stochastic Reaction Dynamics

In an earlier study [10], we presented the formulation of a cell population master equation (CPME) that describes cell population dynamics and takes into account the major sources of heterogeneity: stochasticity of intracellular reactions, DNA-duplication, cell division, and random partitioning of species contents into the two daughter cells. That formulation also takes into account cell growth and respects the discrete nature of the molecular contents and cell numbers. Our approach assumes that each cell of a population can be completely described by a state vector **z **= (**X**, *V*) where **X **is a vector with n entries for species copy numbers and *V *is the volume of the cell. Additional morphometric characteristics like cell membrane area or length can be easily incorporated into our framework. We also classify the chemical species into non-chromosomal DNA species and chromosomal DNA species that may exist in various states.

This CPME describes the evolution of the probability of finding at time *t *the cell population having ν cells that exist in states (**X**_1_, *V*_1_),..., (**X***_i_*, *V_i_*),..., (**X**_ν_, *V*_ν_). If we denote this probability by *J*_ν_[(**X**_1_, *V*_1_),..., (**X***_i_*, *V_i_*),..., (**X**_ν_, *V*_ν_); *t*], the analogue of the Janossy density used in the continuous population balances [25], the CPME becomes [10]:(1)

where the terms *F_R_*, *F_S_*, *F_G _*and *F_D _*describe, respectively, the stochastic dynamics of intracellular reactions, DNA duplication, cell growth, and cell division. The form of these terms is presented in Eq. 6 of Additional file 1. Our earlier publication [10] provides the details about the development of the CPME and the Monte Carlo algorithm that simulates the stochastic processes it describes (this information is also included in Section S1 of Additional file 1 for convenience). Here, we just note that the partitioning of cellular volumes upon division is governed by a symmetric beta distribution. Further, the partitioning of non-chromosomal DNA species follows the binomial distribution whereas for chromosomal DNA species partitioning is governed by a multivariate hypergeometric distribution. These choices result in division events where the daughters may have different sizes and species contents; however, on average any daughter inherits half the contents and has half the size of the mother cell. In a more general setting, it is possible to use sums of non-symmetric beta distributions to describe asymmetric (biased) division (see Section S2 in Additional file 1). In this case, one daughter will consistently inherit more of the mother's molecular content.

### Population Model with Deterministic Reaction Dynamics

The extension of our approach to the case where reactions are deterministic is straightforward and is presented in detail in Section S1 of Additional file 1. For this model, the intracellular species contents are assumed to be continuous vector variables that vary according to reactions described by a system of ordinary differential equations (ODEs). The processes of cell growth and DNA duplication are treated in a similar manner as before, while a modified partitioning function is used here to account for the fact that the chemical species are no longer discrete entities. The modified CPME equation then describes a piecewise deterministic Markov process (a general framework appears in ref. [26]) and takes the form:(2)

where the terms , *F_S_*, *F_G _*and  describe, respectively, the deterministic dynamics of intracellular reactions and cell growth, and stochastic DNA duplication and cell division. Section S1 of Additional file 1 provides the details for the derivation of Eq. 2, which is essentially the deterministic limit of Eq. 1 for all species being present in large numbers inside the cell. In this limit, the stochastic fluctuations due to the individual reaction events are suppressed. As a consequence, reaction dynamics become deterministic (see Eqs. 11, 14 and pertinent discussion in ref. [6]) and the partitioning of species is now governed by Dirac delta distributions (see Section S3 of Additional file 1).

### Structured Continuum Model: a Lumped Description of Cell Population Dynamics

Since the simulation of detailed population models can be computationally expensive, one often resorts to simpler models. In this study, we will use and evaluate the structured continuum model formulation attributed to Fredrickson [9]. This model essentially lumps the volume occupied by the cells into a continuous and expanding (due to cell growth) biotic phase. Thus, the structured continuum model consists of transient mass balance equations written for species concentrations, assuming deterministic reaction dynamics and taking into consideration the dilution effect due to cell growth. A general continuum model can thus be written as a set of ordinary differential equations:(3)

where [*X_i_*] is the average intracellular concentration (an intensive quantity) of species *i *over the entire cell population and μ is the average specific growth rate:(4)

### The Artificial *lac *Operon Reaction Network

To simulate the artificial *lac *operon genetic network, we will use the reaction network of Stamatakis and Mantzaris [17]. This is a minimal model that neglects effects such as the σ70 dependence of the *lac *promoter and assumes that only one *lacO *operator is functional; thus, DNA looping is not accounted for. The existence of three operator sites has been previously shown to result in stronger repression and higher sensitivity in induction [27], as well as lower sensitivity to changes in repressor molecules and lower transcriptional noise [28]. Computational studies of mutations and deletions in these operators have been able to successfully reproduce experimental results [29,30].

Here, our intention has been to model an artificial *lac *operon that can be constructed in the lab rather than the natural *lac *operon system. This artificial system incorporates the positive feedback from the LacY permease resulting in bistable behavior, and thus, allows us to isolate the contributions of stochasticity and bistability in shaping the behavior of the cell population. A more complicated pathway incorporating the aforementioned interactions would be intractable within the cell population frameworks that we have developed, and it is not within the scope of this work to investigate these effects.

Table 1 lists the reactions of this network and presents the corresponding propensity functions needed for the term *F_R _*of the CPME (1) with stochastic reaction dynamics. Table 2 presents the rate equations needed for the term  of the modified CPME (2) with deterministic reaction and growth dynamics and stochastic DNA-duplication and division. The transient mass balances that constitute the structured continuum model (Eqs. 3 and 4 above) are presented in Section S4 of Additional file 1. Note again that the variables of the structured continuum model are intensive quantities that correspond to the average intracellular concentrations of a chemical species over the entire population that is treated as a lumped and expanding biotic phase [9].

**Table 1:** Reactions and Propensity Functions for the Stochastic *lac *Operon Model

Reaction		**Propensity Function**^**1, 2, 3**^
(1-1)		
(1-2)		
(1-3)		
(1-4)		
(1-5)		
(1-6)		
(1-7)		
(1-8)		
(1-9)		
(1-10)		
(1-11)		
(1-12)		
(1-13)		
(1-14)		
(1-15)		
(1-16)		
(1-17)		
(1-18)		
(1-19)		
(1-20)		
(1-21)		
(1-22)		
(1-23)		
(1-24)		
(1-25)		

^1 ^Variables without brackets denote number of molecules of the corresponding species.

^2 ^All propensity functions have units of min^-1^.

^3 ^Avogadro's number: *N_A _*= 6.0221367·10^14 ^nmol^-1^.

**Table 2:** Rate Equations for the Deterministic *lac *Operon Model

(2-1)						
(2-2)						
(2-3)						
(2-4)						
(2-5)						
(2-6)						
(2-7)						
(2-8)						
(2-9)						
(2-10)						

Finally, Table 3 presents the parameter values of the *lac *operon reaction model, the propensities *a_s _*and *a_d _*appearing in the terms *F_S _*and *F_D _*of Eqs. 1 and 2, the growth rate *g *needed for the term *F_G_*, and the volume-related part of the partitioning PDFs (see Eq. 12 in Additional file 1). The parameter set is consistent with the one used in ref. [17].

**Table 3:** Parameters of the *lac *operon models

Symbol	Value	Units	Description
*R_0,E. coli_*	0.4	μm	*E. coli *radius
*L_E. coli_*	2.3	μm	Representative *E. coli *length
*A_E. coli_*	5.8	μm^2^	*E. coli *membrane area (for the above length)
*V_E. coli_*	1.0	fL	*E. coli *volume (for the above length)
*O_T_*	1	(copy number)	operator molecular content
*k_sMR_*	0.23	nM·min^-1^	*lacI *transcription rate
*k_sR_*	15	min^-1^	LacI monomer translation rate constant
*k_2R_*	50	nM^-1^·min^-1^	LacI dimerization rate constant
*k_-2R_*	10^-3^	min^-1^	LacI dimer dissociation rate constant
*k_r_*	960	nM^-1^·min^-1^	association rate constant for repression
*k_-r_*	2.4	min^-1^	dissociation rate constant for repression
*k_dr1_*	3·10^-7^	nM^-2^·min^-1^	association rate constant for 1^st ^derepression mechanism
*k_-dr1_*	12	min^-1^	dissociation rate constant for 1^st ^derepression mechanism
*k_dr2_*	3·10^-7^	nM^-2^·min^-1^	association rate constant for 2^nd ^derepression mechanism
*k_-dr2_*	4.8·10^3^	nM^-1^·min^-1^	dissociation rate constant for 2^nd ^derepression mechanism
*k_s1MY_*	0.5	min^-1^	*lacY *transcription rate constant
*k_s0MY_*	0.01	min^-1^	leak *lacY *transcription rate constant
*k_sY_*	30	min^-1^	*lacY *translation rate constant
*k_p_*	0.12	nM^-1^·min^-1^	LacY-inducer association rate constant
*k_-p_*	0.1	min^-1^	LacY-inducer dissociation rate constant
*k_ft_*	6·10^4^	min^-1^	TMG facilitated transport constant
*h_t_*	1.55·10^-6^	dm·min^-1^	TMG passive diffusion permeability constant
λ*_MR_*	0.462	min^-1^	*lacI *mRNA degradation constant
λ*_MY_*	0.462	min^-1^	*lacY *mRNA degradation constant
λ*_R_*	0.2	min^-1^	LacI monomer degradation constant
λ*_R2_*	0.2	min^-1^	LacI dimer degradation constant
λ*_Y_*	0.2	min^-1^	LacY degradation constant
λ*_YIex_*	0.2	min^-1^	LacY-inducer degradation constant
λ*_I2R2_*	0.2	min^-1^	repressor-inducer degradation constant
*g*	0.0231	(min^-1^)	cell growth rate parameter
*n_d_*	25	(dim/less)	division propensity sharpness exponent
*V_d,crit_*	15	(fL)	critical volume for division
*q*	80	(dim/less)	beta distribution sharpness exponent
*n_s_*	25	(dim/less)	DNA duplication propensity sharpness exponent
*V_s,crit_*	10	(fL)	critical volume for DNA duplication

There is considerable uncertainty for some parameters. For instance, the equilibrium constant for repression, k_-r_/k_r _is in the range of 10^-13 ^to 10^-11 ^M [31-33]. The half-life for dissociation of operator DNA fragments from the repressor has been reported as 30 ~ 49 s [31,34]; thus, we have taken the dissociation rate k_-r _= 2.4 min^-1 ^and k_r _= 960 nM^-1^·min^-1 ^which results in k_-r_/k_r _= 2.5·10^-12 ^M. This value for k_r _turns out to be much higher than the experimentally measured one of 0.6 nM^-1^·min^-1^, which is deduced by the 59 s time needed for a repressor to find an operator [35], assuming *V_E.coli _*= 10^-15 ^L. Yet, simulations of the single cell deterministic model with this value of k_r _produce quantitatively identical bifurcation structures with the nominal parameter set, provided that the value of k_-r _has been adjusted to keep the thermodynamic constant for repression the same. Since the timescale for repression will be different in this case, the single cell stochastic model is expected to exhibit bistability for different induction levels. Previous work [17] showed that slower repressor-operator association and dissociation results in wider bistable regions. In general, however, we expect all qualitative features reported here, to be valid for slower repressor-operator dynamics as well.

For the *lac *operon networks whose parameters are presented in the previous Tables, the propensity functions and rate expression associated with free thiomethyl beta-D-galactoside (TMG) transport require knowledge of the cell membrane area *A_E.coli_*. The latter can be calculated assuming that the *E. coli *cells consist of a cylindrical segment with length *w *and radius *R_0 _*and two hemispherical caps of radius *R_0 _*at both ends. Then the overall length of the cell *L *is equal to *w *+ 2·*R_0_*. Since the diameter of *E. coli *changes only about 8% or less during one division cycle [36], we neglect this change as insignificant in comparison to the two-fold length change of the bacterium. Thus, we assume that *R_0 _*is fixed in the following expression for the volume:(5)

The cell membrane area can be then calculated as a function of the volume (for given *R_0_*):(6)

## Results and discussion

### Comparison of Deterministic and Stochastic Reaction Dynamics

#### Deterministic Reaction Dynamics: Phenotypic Distributions and Statistics

The *lac *operon system is well known for its ability to exhibit bistable behavior when artificial inducers are used [37]. In our model bistability arises from the positive feedback that LacY exerts on its own expression. Other studies suggest that DNA loop structures also play a key role in the bistable behavior of the *lac *operon system [refer to [38,39]]. In this work, however, we focus solely on the aforementioned autocatalytic architecture.

**Figure 2:**
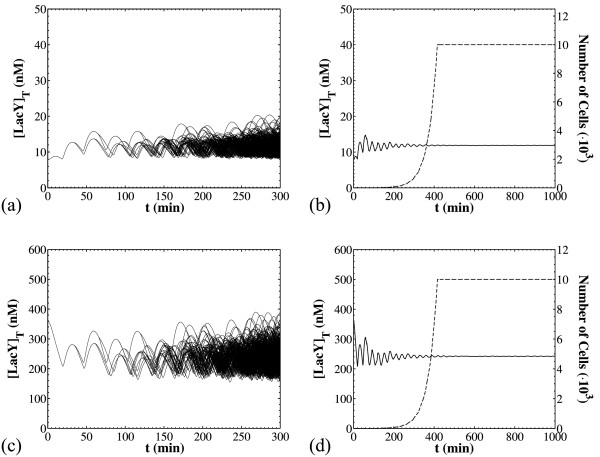
**Simulation results from the CPME model with deterministic reaction dynamics**. The simulations for panels (a) and (b) start with a single cell close to the off-state, while the simulations for panels (c) and (d) start with a single cell close to the on-state. [*I_ex_*] = 24 μM for all simulations and all other parameters as in Table 3. Panels (a) and (c): Transients for all the cells in the corresponding populations. Panels (b) and (d): The solid lines show the dynamics of the population mean for the total LacY concentration for the simulations of panel (a) and (c) respectively. The dashed lines indicate the total number of cells for the corresponding simulations. The constant number technique is used for times greater than 415 min (when *N_cellsmax _*= 10^4^).

Thus, Figure 2 demonstrates the key behavior resulting from this architecture by presenting two representative transient simulations with the modified CPME of Eq. 2. For both simulations, the extracellular inducer TMG concentration is [*I_ex_*] = 24 μM and the only difference is in the initial conditions. In panels (a) and (b), the population is initialized with a single cell having species concentrations close to the off state of the *lac *operon switch. Panel (a) shows the total LacY concentrations, LacY_T_, for every cell in the population versus time. Notice that all offspring remain close to the off state of the *lac *operon switch. Also, the concentrations of the mother and daughter cells are the same at the time of division (Eq. 18 of Additional file 1), and thus, each division appears as a bifurcation in this plot. For example, the first division occurs at 33.8 min. Panel (b) shows the dynamics of the population average for LacY_T_, and the number of cells considered in the population (*N_cellsmax _*= 10^4^, a value reached at 415 min). On the other hand, panels (c) and (d) pertain to the case where the population is initialized with a single cell having species concentrations close to the on-state of the *lac *operon switch. For this case, all offspring remain close to the upper state (panel c), and so does the population average plotted in panel (d).

The oscillations shown in Figure 2 are due to the fluctuations of the promoter concentration during growth and DNA duplication. In particular, at the beginning of the life cycle of a newborn cell, a single promoter exists and the cell has a small volume. Thus, the promoter concentration is high and drops as the cell grows. Right after the DNA duplication event, the concentration of the promoter doubles and subsequently drops again as the cell continues to grow up to the point it divides. Upon cell division, the promoter concentration could also change if division is not symmetric, and thus the daughters inherit half the promoter content but they have volumes that are not equal to *V_mother_*/2. Of course, the concentrations of all other species (that are modeled deterministically) remain the same during DNA duplication and cell division (see section S3 in Additional file 1).

**Figure 3:**
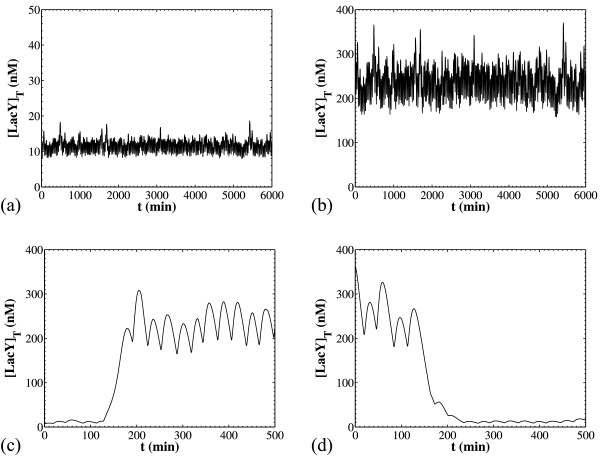
**Attracting states and transitions in the CPME model with deterministic reaction dynamics**. [*I_ex_*] = 24 μM for all simulations and all other parameters as in Table 3. Panels (a) and (b): Dynamics for two cell chains that start with a cell initialized close to (a) the off-state and (b) the on-state. Panel (c): Transition from the off- to the on-state. Cell was initialized close to the off-state. A division occurs at t = 127 min. We impose *V_daughter1_*/*V_mother _*= 0.15 for this division and follow the smaller daughter cell. This is the only intervention throughout the simulation. The cell reaches the on-state and remains there. Panel (d): Transition from the on- to the off-state. For this simulation and just after a DNA duplication occurring at t = 113 min, the division time is set to 50 min. An arbitrary daughter is followed, which eventually reaches the off-state.

The results of Figure 2 may give the impression that it is impossible for a cell to switch between the two states. Furthermore, single cell simulations (*N_cellsmax _*= 1) that follow only one daughter at each division show that even for large simulated times (6000 min ≈ 4 days), no transitions between the upper and the lower attracting state were observed (Figure 3a, b). This behavior can be attributed to the assumption of deterministic reaction dynamics. Thus, in the absence of intrinsic noise it might appear that noise induced transitions are impossible. This assertion is not true, however, because other sources of noise exist in our system. As a matter of fact, a transition from the off to the on state can be achieved just by means of an extremely asymmetric division (see Figure 3c and its legend for details). Such a division results in high DNA concentrations in one daughter (since DNA is partitioned equally). Consequently, the existing free LacY is able to turn on the *lac *operon in that cell, thereby allowing it to reach the upper attracting vicinity. In addition, the opposite transition can also be achieved by means of a delayed cell division (see Figure 3d and its legend for details). The prolonged cell growth after DNA duplication results in low concentrations for the operator, thereby decreasing LacY production rates. Consequently, the autocatalytic feedback weakens and the *lac *operon switches to the low state.

For the simulations of Figure 3c and Figure 3d we have biased the process in order to observe such transitions. Still, we must emphasize the fact that we did not impose conditions that are totally unrealistic. Both extremely asymmetric divisions and long delays in division are possible, albeit highly improbable. Thus, they are not observed within the reasonable simulated time intervals of 6000 min. These observations are in line with results obtained with the models of Vilar et al. [4] and van Hoek and Hogeweg [13], which also predicted no switching between the two equilibrium states, namely, induced and uninduced.

#### Effect of Stochasticity on Cell Population Behavior

Having analyzed the case where reaction dynamics follow deterministic laws, we will now investigate the case of stochastic reaction occurrence since the small copy numbers of molecules encountered in this system are expected to result in significant intrinsic stochasticity.

**Figure 4:**
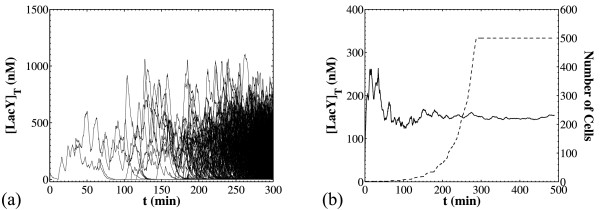
**Simulation results from the CPME model with stochastic reaction dynamics**. [*I_ex_*] = 24 μM for all simulations and all other parameters as in Table 3. Panel (a): Timecourses of all cells in the population. Panel (b): The population average from a batch of 20 simulations with *N_cellsmax _*= 500 in each batch (solid line) and the number of cells in one of the batches (dashed line).

Stochasticity in reaction occurrence results in transcriptional and translational bursts, as shown in panel (a) of Figure 4 that depicts the timecourses of the cells in a population, that is, all daughter cells originating from a single cell. Thus, each cell exhibits noise-induced transitions between states with high and low total LacY contents. We previously saw that in the case of deterministic reaction dynamics, such transitions were possible but extremely rare and were generated either by strongly unequal partitioning or long delays in cell division. Thus, the frequent noise-induced transitions observed in the present case are novel and genuine outcomes of the stochastic nature of reaction occurrence. Furthermore, panel (b) shows the temporal evolution of the cell population average, in which we observe that the cell population average reaches a plateau and fluctuates around an equilibrium point. Note that these fluctuations are a consequence of the finite sample size (low value for *N_cellsmax _*= 500). If we were to simulate without any restriction on the sample size, any fluctuations in the cell population average would eventually disappear. Also note that the population average appears to equilibrate faster in the case of stochastic (Figure 4b) versus that of deterministic (Figure 2, panels b and d) reaction dynamics.

**Figure 5:**
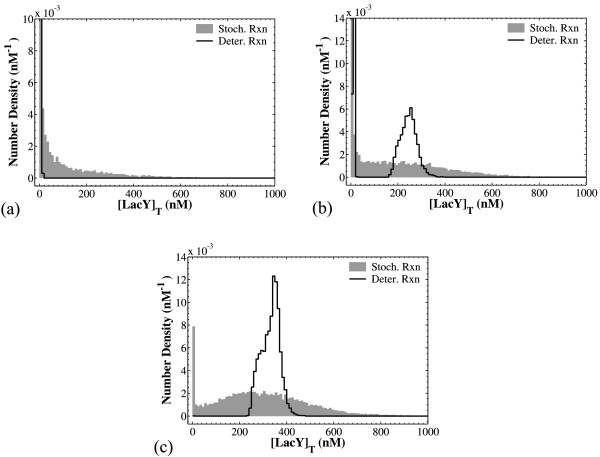
**Comparison of the NDFs computed with deterministic versus stochastic reactions**. NDFs computed with deterministic dynamics are marked as "Deter. Rxn" whereas the ones with stochastic reaction occurrence are marked as "Stoch. Rxn". For all deterministic simulations, the cell population was initiated with 20 cells and *N_cellsmax _*= 10^4^. For all stochastic simulations batches of 20 simulations were run with *N_cellsmax _*= 500. In all cases sampling was performed at t = 300 min. Panel (a): [*I_ex_*] = 10 μM. Panel (b): [*I_ex_*] = 24 μM. Panel (c): [*I_ex_*] = 50 μM. All other parameters as in Table 3.

We have just discussed the effects of stochasticity on the transient behavior of the cells as well as the cell population average. In order to characterize the cell population dynamics, however, one needs to know the entire number density function (NDF) which expresses the number of cells that exist in states (**X**, *V*) and (**X**, *V*+*dV*) (see section 7.1 in ref. [25]). Figure 5 compares the NDFs of the two models, both accounting for stochastic division and DNA duplication, but incorporating deterministic versus stochastic reaction occurrence, thereby revealing the effect of the latter on population heterogeneity.

For low extracellular TMG concentrations, [*I_ex_*], stochasticity creates a heavy tail in the NDF, whereas the simulation with deterministic reactions exhibits a narrow peak in the total LacY concentration (panel a). This heavy tail can be attributed to the autocatalytic mechanism present in the *lac *operon system, an assertion that could be used as a starting point for formulating experimentally testable hypotheses. Heavy tails have also been observed in the absence of positive feedback [40]. In that case heavy tails result from the additive noise due to partitioning of species, as well as due to the exponential evolution of protein number during a division cycle, when protein numbers are large, or the fluctuations in protein production rate, for small protein numbers. These effects exist in our model as well; note, however, that in this study we observe heavy tails in the intensive quantities (species concentrations) as opposed to the extensive quantities (number of protein molecules) of ref. [40]. Since the increase of the protein numbers inside the cell is accompanied by an increase in volume, focusing on the concentration would offset the effect of cell growth. Further, heavy tails have been observed for the single cell *lac *operon model in the absence of growth [17], further supporting our assertion that this behavior is due to the autocatalytic dynamics of the system.

For intermediate inducer concentrations [*I_ex_*], the deterministic reaction NDF is clearly bimodal, in contrast to that for stochastic reaction, which exhibits a much less prominent higher mode (panel b). Thus, intrinsic noise in this case suppresses bistability; yet, a heavy tail indicative of the positive feedback dynamics is still observed. Note that intrinsic noise does not always result in the suppression of bistability: in a previous study pertaining to the single cell level [17] it was shown that intrinsic stochasticity alone can also result in extending the bistable region to parameter values for which a deterministic model would predict monostable behaviour. Finally, for high [*I_ex_*] the NDF for deterministic reaction is unimodal (panel c). However, the NDF for stochastic reaction appears bimodal, with a sharp peak at total LacY concentration equal to zero and a wide peak at high [Y]_T_. In this case, stochasticity in reaction occurrence appears to extend the region where the NDF is bimodal and widen the upper mode of the distribution.

In all cases (Figure 5), the heterogeneity exhibited by the population model with stochastic reaction occurrence is significantly larger than that exhibited by the model with deterministic reaction occurrence. This observation agrees with previous results by van Hoek and Hogeweg [13], whose model also predicted that, in the absence of spatial and genetic heterogeneity, the population variability exhibited by the stochastic simulation is much larger than that of the deterministic one.

### Structured Continuum Model for Deterministic Reaction Dynamics

Simulating in detail the intra- and inter-cellular processes at the population level is computationally demanding. Therefore, it is natural to ask whether one can adequately predict the dynamics of the population average with the use of a lumped model that neglects heterogeneity. For this purpose, we will use the structured continuum model formulation [9] that treats all cells as a lumped and expanding (due to cell growth) biotic phase. Section S4 in Additional file 1 presents all the transient mass balances that constitute the structured continuum model defined by Eqs. 3 and 4. In our case, μ is equal to *g *(Eq. 4) because we have assumed exponential growth, which implies a constant average specific growth rate. Note again that these balances are written for the average intracellular concentrations (intensive quantities) of the chemical species of interest over the entire cell population. The structured continuum model is valid for all times. The assumption here is that the concentrations in any cell at any time remain close to the concentrations predicted by this continuum model. Note that we did not include the dilution effect for the operator species O, since the DNA duplication process continuously regenerates O.

Simulations with the structured continuum model require estimates for the average membrane area over the volume 〈*A*/*V*〉 and the average total operator concentration 〈[*O*]*_T_*〉. These average quantities can either be obtained directly from the population simulation or estimated if we know approximately the sizes at which the cells duplicate their DNA or divide.

The average ratio of area over volume 〈*A*/*V*〉 can be estimated by assuming that a newborn cell with volume 1/2·*V_d,crit _*divides when it reaches *V_d,crit_*. Since the cell is growing exponentially and the *A*/*V *is known from Eqs. 5 and 6:(7)

Therefore, the average 〈*A*/*V*〉 can be calculated over the time course of a birth-division cycle using the first mean value theorem for integration:(8)

Using the parameters of Table 3, this estimated average equals 5.69 μm^-1^. Cell population simulations give an average equal to 5.76 μm^-1^. Thus, using these heuristic arguments, we estimated the average ratio 〈*A*/*V*〉 with a remarkably low error (1.2%).

To estimate the average operator concentration 〈[*O*]*_T_*〉, let us assume that a newborn cell with volume 1/2·*V_d,crit _*duplicates its DNA when it reaches *V_s,crit _*and divides when it reaches *V_d,crit_*. Since the cell is growing exponentially and the initial operator content is 1 copy before duplication:(9)

Using the parameters of Table 3, we obtain an estimate of the average operator concentration equal to 2.40 nM. Cell population simulations give an average equal to 2.48 nM (or 3.2% error).

**Figure 6:**
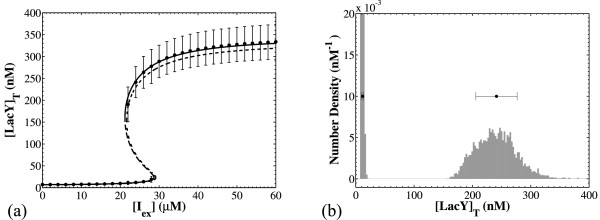
**Comparison of the average stationary behavior of the CPME model that incorporates deterministic reaction dynamics with the steady state of the structured continuum model**. [*I_ex_*] = 24 μM for all simulations and all other parameters as in Table 3. Panel (a): Predictions from the cell population model agree with those from the structured continuum model when the average values for *A*/*V *and [*O*]*_T _*are used. For the solid S-shaped curve, the average values for *A*/*V *and [*O*]*_T _*were taken from the population simulations. For the dashed curve, the average values were estimated using Eqs. 8 and 9. Panel (b): a representative bimodal NDF and the corresponding averages and standard deviation.

Panel (a) of Figure 6 compares the results obtained by the structured continuum model to those obtained by the modified CPME with deterministic reaction dynamics (Eq. 22 in Additional file 1). The solid and dashed curves show bifurcation diagrams for the total steady state LacY concentration calculated by the structured continuum model. For the solid curve, the average values for the membrane area over cell volume ratio and the total operator concentration were computed from the cell population simulation. For the dashed curve, these average values were taken to be equal to the estimates of Eqs. 8 and 9. The symbols with the error bars give the average and standard deviation respectively of the modes of the resulting NDFs computed by the modified CPME with deterministic reaction occurrence. Each segment of the error bar is one standard deviation, so the full error bar is equal to two standard deviations. If the NDF is bimodal (as shown, for example, in panel (b) of Figure 6), the two subpopulations are identified and the means and standard deviations for each subpopulation are computed.

The qualitative agreement between the steady states of the structured continuum model and the population averages is excellent. The agreement between the two models is remarkably good when the population-average values for *A*/*V *and [*O*]*_T _*are used in the structured continuum model. When we use the estimated values from Eqs. 8 and 9, the total LacY concentration at maximal induction is slightly underestimated due to the underestimation of the average total operator concentration. Thus, the level of agreement between the steady states of the structured continuum model and the averages of the population model depends on how good the estimates for the average cell characteristics are.

The above results pertain to the time invariant behaviors of the cell population. It is interesting, however, to also compare the dynamical behavior of the structured continuum and the cell population model.

**Figure 7:**
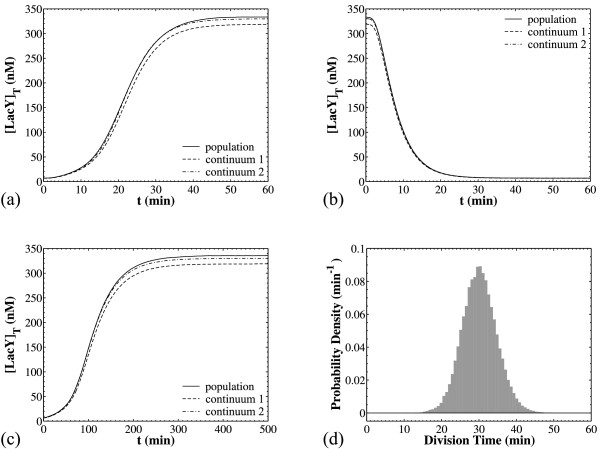
**Comparison of the dynamical behavior of the CPME model that incorporates deterministic reaction dynamics with that of the structured continuum model**. Parameters as in Table 3 unless otherwise noted. Panels (a, b): Transient dynamics of the population mean for the switching from the low ([*I_ex_*] = 0 μM) to the high state ([*I_ex_*] = 60 μM) (panel a) and conversely (panel b). For the dashed curve (continuum 1), the average values for [*O*]*_T _*and *A*/*V *were calculated from Eqs. 8 and 9, and for the dash-dotted curve (continuum 2) they were taken from the population simulations. Panel (c): As in panel (a) but with *k_s0MY _*= 0.025 min^-1^, *k_s1MY _*= 0.0005 min^-1^, λ*_MY _*= 0.001155. Panel (d): The distribution of the division times (for comparison with the switching times).

Panels (a) and (b) of Figure 7 show two such comparisons. The solid curves on both panels show the dynamics of the cell population average as predicted by the CPME model with deterministic reaction dynamics. For the run of panel (a), a population simulation is first performed for [*I_ex_*] = 0 μM. At time t = 1000 min, the resulting cell population (*N_cellsmax _*= 10^4^) has equilibrated to the stationary distribution that corresponds to [*I_ex_*] = 0 μM. Then, this population is used as initial condition for a simulation with [*I_ex_*] = 60 μM in order to observe the dynamics of switching from the low to the high state at the population level. This computational procedure mimics the experimental task of inoculating cells to different environment conditions. The dynamics of the opposite switch are simulated for panel (b): first the cells equilibrate at 60 μM TMG and then the population is "transferred" to 0 μM TMG).

The dashed and dash-dotted curves on panels (a) and (b) of Figure 7 present predictions of the structured continuum model. For the dash-dotted curves, the average values for the cellular characteristics 〈*A*/*V *〉 and 〈[*O*]*_T_*〉 were taken from the cell population simulations, while for the dashed curves these values were estimated using Eqs. 8 and 9. For panel (a), the structured continuum models are simulated for [*I_ex_*] = 60 μM, using as initial conditions the steady state concentrations calculated for [*I_ex_*] = 0 μM. The dynamics of the opposite switch are presented in panel (b).

The agreement between the transient behaviors predicted by the structured continuum model and the population model is excellent even in the case where the intracellular dynamics are significantly slower than the proliferation rates of the cell as shown in panels (c) and (d) of Figure 7. Panel (c) shows that for slow *lacY *mRNA dynamics the switching from the low to the high state takes approximately 300 min, which is much longer than the 30 min average doubling time (panel d). Even so, the structured continuum model yields excellent predictions of the population average. Similar observations were previously reported by Vilar et al. [4], who showed that an approach taking into account single cell stochasticity and population level heterogeneity may not be needed, depending on the system studied and the conditions of interest. In particular, Vilar et al. [4] noted that, when the *lac *operon is under the influence of high inducer concentrations, averages taken over independent cells versus the overall population give very similar results.

This agreement between the steady state and the transient behavior of the structured continuum and the cell population models can be explained as follows. In this system, the only coupling between the cells is due to stochastic partitioning that can generate variability but no bias on the total LacY concentration, in the sense that one does not observe consistently higher LacY_T _concentrations in one daughter versus the other. In fact, the two newborn daughters may have different LacY_T _contents, but they always have equal LacY_T _concentrations, which are identical to their mother's LacY_T _concentration just before division. Similarly, unsynchronized DNA duplication and division events also contribute to the observed variability in LacY_T_, but they cannot consistently bias the LacY concentration.

In general, the key to understanding this effect lies in the fact that in the cell population, division results in the removal of an "old" mother cell and the addition of two "young" daughter cells. If the properties of the "old" cells are different than those of the "young" daughters, then this effect results in the properties of the "young" ones being overrepresented in the population. On the other hand, such an effect is absent in a cell chain, where each division results in the removal of a mother cell and the addition of a single daughter, and thus, the properties of both subpopulations are equally represented in the cell chain probability distribution.

Let us now suppose that the property we chose to investigate is a species concentration (intensive variable). The concentrations of mother and daughter cells are on average the same, due to the symmetric properties of the binomial and beta distributions that model division. Consequently, the cell chain and cell population distributions will be practically indistinguishable. On the other hand, if we chose an intensive property to investigate, the two distributions would be different in general, as we have shown in our previous work [Figure 6 in ref. [10]]. This point is rigorously proven for deterministic reaction dynamics in an earlier publication [41], where we establish that a population balance equation transforms into a continuum model for the species concentrations if certain conditions are satisfied. The conditions, in summary, call for equal species concentrations in mother and daughter cells, size-independent intensive reaction rates, size-dependent but concentration-independent growth and division rates and partitioning probability density function. All conditions hold true to a good approximation in our present simulations and they are also expected to be biologically plausible for a range of systems of interest.

These observations contradict previously published results by Mantzaris for different biological systems [12,16,42,43]. In these studies, comparisons of the steady states of structured continuum models with the stationary averages of cell population balances (CPBs) showed that the regions in which the two models exhibit specific types of behavior, such as bistability, were vastly different. These differences were interpreted as effects of cell population heterogeneity. However, we believe that the differences shown by Mantzaris [12,16,42,43] are not genuine effects of heterogeneity, but rather stem from the modeling assumptions employed.

More specifically, these studies incorporated into the CPB single cell models that are written for species concentrations. However, the CPB accepts single cell models written for cellular contents (amounts), which are extensive quantities, and not concentrations which are intensive [9]. One immediate consequence of this fact is by partitioning the concentrations upon cell division one would violate mass conservation. Moreover, the cell volume is not taken into account in any of these studies. Cells in the exponential phase, however, are continuously growing and dividing, thereby changing their volume roughly two-fold during one division cycle. In turn, the change in volume shifts the thermodynamic equilibrium point and also affects the dynamics if multi-molecular reactions are present in the reaction scheme, which is the case in all of the systems considered in these studies [12,16,42,43]. Therefore, neglecting the change of the cell volume as time progresses changes creates *a priori *an inconsistency between the single cell expressions incorporated into the population balance and the structured continuum model.

Furthermore, some of these studies used unequal partitioning to artificially generate complex behavior, such as oscillations in a reaction network with 0^th ^and 1^st ^order reactions. However, the asymmetry in *E. coli *division is negligible: cells may stochastically divide in two unequal daughters, but consistent generation of one large and one small daughter has not been experimentally observed. In fact, it has been shown that the distribution of daughter cell sizes has a mean corresponding to equal partitioning and a small coefficient of variation [44,45].

### Simulation of Cell Chains for Stochastic Reaction Dynamics

Given the high computational expense of simulating the population model with stochastic reaction dynamics, we pose the question of whether there is a simpler method for obtaining good approximations for the distributions of phenotypic characteristics. In contrast to the case of deterministic reaction dynamics, we cannot use a continuum model for comparison purposes here. The main reason is that in chemical systems far from the thermodynamic limit, the size influences noise strength. In the case of stochastic reaction occurrence, therefore, growth results in dilution of molar contents and also suppression of stochastic fluctuations. Consequently, we cannot create a lumped model that accounts for growth with just the incorporation of a dilution term as was done in the deterministic case.

However, we can take a different approach. We have already observed that cell population dynamics emerge from single cell behavior and that, on average, the two daughters share the same concentrations as their mother cell. Therefore, instead of tracking the NDF in a cell population, we could compute the PDF in a cell chain. Simulation of a cell chain tracks only one daughter after each division event. Thus, instead of focusing on the expected number of cells of the population that exist in state **z**, we turn our attention to the probability of finding a single cell of the cell chain at state **z**. Note, however, that we will be comparing the PDF and NDF of intensive quantities, in particular the total LacY concentration.

**Figure 8:**
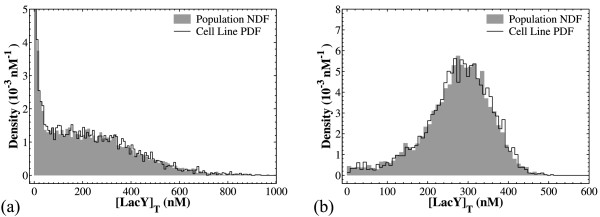
**Comparisons of cell chain probability distribution functions with cell population NDFs**. Panel (a): For the population distribution, a batch of 20 simulations was run with *N_cellsmax _*= 500, [*I_ex_*] = 24 μM and the nominal parameter set (Table 3) and sampled at t = 500 min. For the cell chain simulation, t_final _= 10^5 ^min and samples were taken periodically in time with Δ*t *= 10 min. Panel (b): as in panel (a) but with 100-fold faster *lacY *transcription (*k_s1MY _*= 50 min^-1^, *k_s1MY _*= 1 min^-1^) and slower translation (*k_sY _*= 0.3 min^-1^). The simulation batch consisted of 20 simulations and was sampled at t = 250 min. The cell line was tracked for 10^5 ^min of simulated time.

A comparison of the cell population NDF with the single cell PDF for [Y]_T _shows a remarkable agreement between the two (Figure 8). Such an agreement is obtained for different parameter sets that generate dissimilar distributions. Note that this comparison pertains to stationary conditions and refers to intensive quantities (namely the total LacY concentration). Essentially, this comparison shows that under the assumptions used to build the models, the cell chain behavior is indicative of the population behavior. Thus, we can successfully predict the stationary NDF of the concentrations just by simulating a cell chain, which is computationally less expensive than simulating the cell population.

## Conclusions

This study generalized the deterministic and stochastic single cell *lac *operon models of an earlier publication [17] using two cell population frameworks that account for stochastic and deterministic dynamics of the biochemical reactions. We subsequently performed simulations to investigate the behavior of the cell population and demonstrated the effect of stochasticity in reaction dynamics. We concluded that this source of stochasticity can amplify population heterogeneity by introducing heavy tails to the phenotypic distributions and can create or destroy bimodality.

We also carried out a systematic comparison of predictions obtained by a structured continuum model and a detailed cell population model with deterministic reaction dynamics. These comparisons showed that the structured continuum model gives satisfactory results for the average LacY_T _concentration of the population, even in the case where the reaction dynamics are much slower than the proliferation dynamics. This agreement between the two models was attributed to the similar intensive properties (such as species concentrations) between mother and daughter cells.

Finally and in the case of stochastic reaction dynamics, we demonstrated that by simulating the dynamics of a cell chain we can obtain very accurate approximations of the cell population dynamics. The PDFs obtained by cell chain simulations for the LacY_T _concentration were in excellent agreement to the cell population NDF computed with the Monte Carlo algorithm implementing the CPME of Eq. 1.

Our study shows that for cell populations in which the cells interact weakly, through division only, it is possible to accurately model and explain the population behavior in terms of the single cell dynamics. For such systems, the key parameters for describing the behavior of the population are the kinetic constants of the underlying pathway of interest, and the physiological functions that express the single cell growth rate, DNA-duplication and division propensity, as well as the partitioning mechanism. Intrinsic noise can be inherently accounted for, once the cell size has been specified and thus no additional parameters are needed for this purpose. Such a description is expected to be of great importance in bioinformatics studies focusing on population variability, since, for cells interacting though division only this variability can be explained in terms of a limited number of parameters.

Finally, deviations between the experimentally observed population dynamics and the behavior predicted by our framework may indicate the presence of more complex effects that are not accounted for in this framework. For instance, the cells could be non-isogenic, or coupled with strong interaction mechanisms. Another source of complexity is the existence of multiple compartments in the cell, which would invalidate the assumption of a single well-mixed intracellular space. Such effects would have to be incorporated to the framework in order to obtain a more accurate description of the system of interest.

## Competing interests

The authors declare that they have no competing interests.

## Author's Contributions

MS developed the mathematical models and performed the simulations, analyzed the results and drafted the manuscript. KZ conceived of the study, participated in its design and coordination, and refined the manuscript. Both authors read and approved the final manuscript.

## Supplementary Material

Additional file 1 **Supplementary text that includes**: (1) detailed descriptions of the population level modeling frameworks for deterministic and stochastic reaction dynamics; (2) a discussion of asymmetric volume partitioning; (3) the derivations of the partitioning probabilities for population models with deterministic reaction dynamics (4) the structured continuum model simulated in Figure 6 and Figure 7. Click here for file
