# Supplementary material for: Deterministic and stochastic population-level simulations of an artificial lac operon genetic network
Source: BMC Bioinformatics. 2011 Jul 26;12:301. doi: 10.1186/1471-2105-12-301 (PMC3181209; doi:10.1186/1471-2105-12-301)
Supplement: Additional file 1 — Supplementary text that includes: (1) detailed descriptions of the population level modeling frameworks for deterministic and stochastic reaction dynamics; (2) a discussion of asymmetric volume partitioning; (3) the derivations of the partitioning probabilities for population models with deterministic reaction dynamics (4) the structured continuum model simulated in Figure 6 and Figure 7. [file 1471-2105-12-301-S1.PDF]

SUPPLEMENTARY MATERIAL

Deterministic and Stochastic Population Level Simulations of an Artificial *lac* Operon Genetic Network

Michail Stamatakis and Kyriacos Zygourakis

Department of Chemical and Biomolecular Engineering, Rice University, Houston, TX 77005, USA

SECTION S1: POPULATION LEVEL MODELING FRAMEWORKS

Population Model with Stochastic Reaction Dynamics

We assume that each cell of a population can be completely described by a state vector $\mathbf{z} = \left(\mathbf{X}, V\right)$ where $\mathbf{X}$ is a vector with n entries for species copy numbers and V is the volume of the cell. Additional morphometric characteristics like cell membrane area or length can be easily incorporated into our framework.

The chemical species are classified into non-chromosomal DNA species and chromosomal DNA species that may exist in various states. Thus, the species vector can be written as:

$$S = \left\{ \underbrace{S_1, S_2, \ldots, S_n}_{\text{Non-chrom. DNA}}, \underbrace{S_{n+1}, S_{n+2}, \ldots, S_{n+s_1}}_{\substack{\text{Chrom. DNA species 1} \\ \text{in its various states}}}, \ldots, \underbrace{S_{N-s_d+1}, S_{N-s_d+2}, \ldots, S_N}_{\substack{\text{Chromosomal DNAspecies d} \\ \text{in its various states}}} \right\} \tag{1}$$

and the total number of species is defined as:

$$N = n + \sum_{i=1}^{d} s_i \tag{2}$$

where n is the number of non-chromosomal DNA species and d is the number of chromosomal DNA species. The necessity for discriminating between chromosomal and non-chromosomal species comes from the fact that, upon division, chromosomal DNA species are partitioned equally in the two daughters. However, this is not generally true for the other species. Furthermore, each of the chromosomal DNA species $i = 1, 2, \ldots, d$ may exist in s_{i} states. For example an operator may exist in three states: the free state O, the repressed state with one bound repressor molecule RO, or the repressed state with two bound repressor molecules R_2O. Thus for this case,

$$s_1 = 3 \text{ and } \left(S_{n+1}, S_{n+2}, S_{n+3}\right) = \left(O, RO, R_2O\right) \tag{3}$$

The chemical species are assumed to interact according to the following network of *m* reactions with *N* participating species S_i:

$$\left\{\sum_{i=1}^{N} \alpha_{ij} S_i \xrightarrow{k_j} \sum_{i=1}^{N} \beta_{ij} S_i \right\}_{j=1}^{m} \tag{4}$$

Similarly, DNA duplication can be represented as a reaction, where all chromosomal DNA species in any state are doubled:

$$\left\{\left\{S_{\eta_i + j} \longrightarrow S_{\eta_i + j} + S_{\eta_i}\right\}_{j=1}^{s_i}\right\}_{i=1}^{d} \quad \text{where:} \quad \eta_i = n + \sum_{j=1}^{i-1} s_j + 1 \tag{5}$$

Our CPME describes the evolution of the probability of finding at time *t* the cell population having ν cells that exist in states $\left(\mathbf{X_1}, V_1\right), \left(\mathbf{X_2}, V_2\right), \ldots, \left(\mathbf{X_\nu}, V_\nu\right)$. We denote this probability by $J_\nu\left(\left(\mathbf{X_1}, V_1\right), \ldots, \left(\mathbf{X_i}, V_i\right), \ldots, \left(\mathbf{X_\nu}, V_\nu\right); t\right)$, the analogue of the Janossy density used in the continuous population balances [1]. Then, the final form of the CPME becomes [2]:

$$\frac{\partial}{\partial t} J_\nu\left(\left(\mathbf{X_1}, V_1\right), \ldots, \left(\mathbf{X_i}, V_i\right), \ldots, \left(\mathbf{X_\nu}, V_\nu\right); t\right) = F_R + F_S + F_G + F_D$$

where

$$F_R = \sum_{\varsigma=1}^{\nu} \sum_{j=1}^{m} \Big[a_j\left(\mathbf{X_\varsigma} - \mathbf{v_j}, V_\varsigma\right) \cdot J_\nu\left(\left(\mathbf{X_1}, V_1\right), \ldots, \left(\mathbf{X_\varsigma} - \mathbf{v_j}, V_\varsigma\right), \ldots, \left(\mathbf{X_\nu}, V_\nu\right); t\right) + \\ - a_j\left(\mathbf{X_\varsigma}, V_\varsigma\right) \cdot J_\nu\left(\left(\mathbf{X_1}, V_1\right), \ldots, \left(\mathbf{X_\varsigma}, V_\varsigma\right), \ldots, \left(\mathbf{X_\nu}, V_\nu\right); t\right) \Big]$$

$$F_S = \sum_{\varsigma=1}^{\nu} \Big[a_s\left(\mathbf{X_\varsigma} - \mathbf{v_s}, V_\varsigma\right) \cdot J_\nu\left(\left(\mathbf{X_1}, V_1\right), \ldots, \left(\mathbf{X_\varsigma} - \mathbf{v_s}, V_\varsigma\right), \ldots, \left(\mathbf{X_\nu}, V_\nu\right); t\right) + \\ - a_s\left(\mathbf{X_\varsigma}, V_\varsigma\right) \cdot J_\nu\left(\left(\mathbf{X_1}, V_1\right), \ldots, \left(\mathbf{X_\varsigma}, V_\varsigma\right), \ldots, \left(\mathbf{X_\nu}, V_\nu\right); t\right) \Big]$$

$$F_G = -\sum_{\varsigma=1}^{\nu} \frac{\partial}{\partial V_\varsigma} \Big[g\left(\mathbf{X_\varsigma}, V_\varsigma\right) \cdot J_\nu\left(\left(\mathbf{X_1}, V_1\right), \ldots, \left(\mathbf{X_\varsigma}, V_\varsigma\right), \ldots, \left(\mathbf{X_\nu}, V_\nu\right); t\right) \Big]$$

and

$$F_D = 2 \cdot \sum_{\varsigma=1}^{\nu-1} \sum_{\theta=\varsigma+1}^{\nu} a_d\left(\mathbf{X_\varsigma} + \mathbf{X_\theta}, V_\varsigma + V_\theta\right) \cdot h\left(\mathbf{X_\varsigma}, V_\varsigma \middle| \mathbf{X_\varsigma} + \mathbf{X_\theta}, V_\varsigma + V_\theta\right) \cdot \\ \cdot J_{\nu-1} \cdot \left(\left(\mathbf{X_1}, V_1\right), \ldots, \left(\mathbf{X_\varsigma} + \mathbf{X_\theta}, V_\varsigma + V_\theta\right), \ldots, \left(\mathbf{X_{\theta-1}}, V_{\theta-1}\right), \left(\mathbf{X_{\theta+1}}, V_{\theta+1}\right), \ldots, \left(\mathbf{X_\nu}, V_\nu\right); t\right) + \\ - \sum_{\varsigma=1}^{\nu} a_d\left(\mathbf{X_\varsigma}, V_\varsigma\right) \cdot J_\nu\left(\left(\mathbf{X_1}, V_1\right), \ldots, \left(\mathbf{X_\varsigma}, V_\varsigma\right), \ldots, \left(\mathbf{X_\nu}, V_\nu\right); t\right) \tag{6}$$

The term F_R of Eq. 6 comes from the stochastic treatment of intracellular reactions, where $a_j(\mathbf{X},V)$ denotes the propensity with which a reaction may occur in some cell with state $(\mathbf{X},V)$ and is given by

$$a_j(\mathbf{X},V) = k_j \cdot N_A \cdot V \cdot \prod_{i=1}^{N} \frac{\alpha_{ij}\,!}{(N_A \cdot V)^{\alpha_{ij}}} \cdot \binom{X_i}{\alpha_{ij}} \tag{7}$$

where N_A is Avogadro's number.

The term F_S of Eq. 6 comes from DNA duplication where $a_s(\mathbf{X},V)$ is the corresponding propensity function that is volume dependent and given by:

$$a_j(\mathbf{X},V) = \left(\frac{V}{V_{s,crit}}\right)^{n_s} \cdot \delta_{\sum_{j=n+1}^{n+s_1} X_j, U_1} \tag{8}$$

The Kronecker delta δ in Eq. 8 is unity when the copy numbers of chromosomal species 1 in any state sum to a nominal pre-duplication copy number U_1. This ensures that duplication is performed only once per cycle, when the chromosomal DNA species have copy number equal to U_1 and the cell volume (size) is close to $V_{s,crit}$. Furthermore, n_s modulates the sharpness of the DNA-duplication mechanism: very high values result in duplication occurring precisely when the cell volume reaches the value $V_{s,crit}$. Lower values result in DNA-duplication occurring randomly when the cell volume is around this critical value.

The term F_G of Eq. 6 describes cell growth as a continuous deterministic process given the species contents and at the single cell level can be represented as an ODE. This formulation assumes exponential single cell growth rate [3] and:

$$\frac{dV}{dt} = g \cdot V \tag{9}$$

Finally, the term F_D of Eq. 6 describes cell division that occurs with propensity given by:

$$a_d(\mathbf{X},V) = \left(\frac{V}{V_{d,crit}}\right)^{n_d} \cdot \delta_{\sum_{j=n+1}^{n+s_1} X_j, 2\cdot U_1} \tag{10}$$

Again, $V_{s,crit}$ is the critical volume that must be approached for the division to occur and n_d modulates the sharpness of the division mechanism.

Since the partitioning of the mother into two daughter cells is random, we need to define the partitioning probability density function (PDF) $h(\mathbf{X_d}, V_d \mid \mathbf{X_m}, V_m)$ that gives the probability that

the state of the daughter cell is $(\mathbf{X_d}, V_d)$ given that the state of the mother was $(\mathbf{X_m}, V_m)$. Our model assumes that volume partitioning is independent of content partitioning and, moreover, that partitioning occurs independently for every species (non-chromosomal or chromosomal DNA). Thus, the partitioning probability takes the form of the following product:

$$h(\mathbf{X_d}, V_d \mid \mathbf{X_m}, V_m) = \beta(V_d \mid V_m) \cdot \prod_{i=1}^{n} b_i\left(X_{d,i} \mid \mathbf{X_m}, V_m, V_d\right) \cdot \prod_{i=1}^{d} c_i\left(\mathbf{X_d^{DNA,i}} \mid \mathbf{X_m}, V_m, V_d\right) \tag{11}$$

The function β describes cell volume partitioning that is assumed to have the form of a symmetric beta distribution:

$$\beta(V_d \mid V_m) = \frac{1}{V_m} \cdot \frac{\Gamma(2 \cdot q)}{\left(\Gamma(q)\right)^2} \cdot \left(\frac{V_d}{V_m}\right)^{q-1} \cdot \left(1 - \frac{V_d}{V_m}\right)^{q-1} \tag{12}$$

where *q* is a parameter controlling the sharpness of the division mechanism. The product of the functions b_i, i = 1,2,…,*n* describes binomial partitioning of the non-chromosomal DNA species with:

$$b_i\left(X_{d,i} \mid X_{m,i}, V_m, V_d\right) = \binom{X_{m,i}}{X_{d,i}} \cdot \left(\frac{V_d}{V_m}\right)^{X_{d,i}} \cdot \left(1 - \frac{V_d}{V_m}\right)^{X_{m,i} - X_{d,i}} \qquad \text{for } i = 1, \ldots, n \tag{13}$$

The chromosomal DNA species require symmetric partitioning but with randomized state. The functions c_i describe partitioning of these species and are given by:

$$c_i\left(\mathbf{X_d^{DNA,i}} \mid \mathbf{X_m}, V_m, V_d\right) = \prod_{j=1}^{s_i} \binom{X_{m,j}^{DNA,i}}{X_{d,j}^{DNA,i}} \cdot \binom{\sum_{j=1}^{s_i} X_{m,j}^{DNA,i}}{\frac{1}{2} \cdot \sum_{j=1}^{s_i} X_{m,j}^{DNA,i}}^{-1} \qquad \text{for } i = 1, \ldots, d \tag{14}$$

The full details about the development of the CPME and the Monte Carlo algorithm that simulates the stochastic processes it describes are given in our earlier publication [2].

Population Model with Deterministic Reaction Dynamics

The extension of our approach to the case where reactions are deterministic is straightforward. The species contents $\mathbf{X_i}$, $i = 1,\ldots,\nu$, are now continuous vector variables that vary according to reactions described by a system of ordinary differential equations (ODEs). Reactions are treated in a similar way as cell growth in the aforementioned stochastic model. Thus:

$$\frac{dX_i}{dt} = r_i\left(\mathbf{X},V\right) = \sum_{j=1}^{m}\left(\beta_{ij}-\alpha_{ij}\right)\cdot k_j\cdot V\cdot\prod_{k=1}^{N}\left(\frac{X_k}{V}\right)^{\alpha_{kj}} \qquad i=1,\dots,N$$

$$\frac{dV}{dt} = g\left(\mathbf{X},V\right) \tag{15}$$

Therefore, the net outflow of probability from state $\left(\nu,\left(\mathbf{X_1},V_1\right),\dots,\left(\mathbf{X}_\varsigma,V_\varsigma\right),\dots,\left(\mathbf{X}_\nu,V_\nu\right)\right)$ due to reactions occurring only in cell ς will be given as:

$$-\nabla_{\mathbf{X}_\varsigma}\cdot\left[\mathbf{r}\left(\mathbf{X}_\varsigma,V_\varsigma\right)\cdot J_\nu\left(\left(\mathbf{X_1},V_1\right),\dots,\left(\mathbf{X}_\varsigma,V_\varsigma\right),\dots,\left(\mathbf{X}_\nu,V_\nu\right);t\right)\right] \tag{16}$$

where $\mathbf{r}$ is the vector with the rate expressions for the production of species x_k, $k = 1,2,\dots,N$ (with the convention that r_k is positive when x_k is produced and negative when x_k is depleted), and the divergence $\nabla_{\mathbf{X}_\varsigma}\cdot$ operates in a vector function $\mathbf{Z}$ as:

$$\nabla_{\mathbf{X}_\varsigma}\cdot\mathbf{Z} \equiv \sum_{i=1}^{N}\frac{\partial Z_i}{\partial X_{\varsigma,i}} \tag{17}$$

For the partitioning function h, the components that express species partitioning can no longer be given by Eqs. 13 and 14 since now the species to be partitioned are not discrete entities. Thus, new expressions must be formulated:

$$b_i\left(X_{d,i}\mid X_{m,i},V_m,V_d\right) = \delta\left(X_{d,i}-\frac{V_d}{V_m}\cdot X_{m,i}\right) \qquad \text{for } i=1,\dots,n \tag{18}$$

$$c_i\left(\mathbf{X}_\mathbf{d}^{\mathbf{DNA,i}}\mid\mathbf{X_m},V_m,V_d\right) = \prod_{j=1}^{s_i}\delta\left(X_{d,j}^{DNA,i}-\frac{1}{2}\cdot X_{m,j}^{DNA,i}\right) \qquad \text{for } i=1,\dots,d \tag{19}$$

Hence, the content for non-chromosomal DNA species is partitioned between the two daughter cells proportionally to each daughter’s volume. For chromosomal DNA species equal partitioning is always occurring. Intuitively, limiting agreement between Eqs. 13-14 and 18-19 respectively is guaranteed by the behavior of the binomial and the hypergeometric distributions, which approach Dirac delta functions at the limit of infinitely many trials. Partitioning of the volume is still taken to follow a symmetric beta:

$$\beta\left(V_d\mid V_m\right) = \frac{1}{V_m}\cdot\frac{\Gamma\left(2\cdot q\right)}{\left(\Gamma\left(q\right)\right)^2}\cdot\left(\frac{V_d}{V_m}\right)^{q-1}\cdot\left(1-\frac{V_d}{V_m}\right)^{q-1} \tag{20}$$

and the full partitioning function is given as previously:

$$h^{*}\left(\mathbf{X_d},V_d \mid \mathbf{X_m},V_m\right) = \beta\left(V_d \mid V_m\right)\cdot\prod_{i=1}^{n} b_i\left(X_{d,i} \mid \mathbf{X_m},V_m,V_d\right)\cdot\prod_{i=1}^{d} c_i\left(\mathbf{X_d^{DNA,i}} \mid \mathbf{X_m},V_m,V_d\right) \tag{21}$$

Hence, the modified CPME equation, which treats species contents as continuous variables and reaction dynamics as deterministic becomes:

$$\frac{\partial}{\partial t} J_\nu\left(\left(\mathbf{X}_1,V_1\right),\ldots,\left(\mathbf{X}_i,V_i\right),\ldots,\left(\mathbf{X}_\nu,V_\nu\right);t\right) = F_R^{*} + F_S + F_G + F_D^{*}$$

where

$$F_R^{*} = -\sum_{\varsigma=1}^{\nu}\nabla_{\mathbf{X}_\varsigma}\cdot\left[\mathbf{r}\left(\mathbf{X}_\varsigma,V_\varsigma\right)\cdot J_\nu\left(\left(\mathbf{X}_1,V_1\right),\ldots,\left(\mathbf{X}_\varsigma,V_\varsigma\right),\ldots,\left(\mathbf{X}_\nu,V_\nu\right);t\right)\right]$$

$$F_S = \sum_{\varsigma=1}^{\nu}\Big[a_s\left(\mathbf{X}_\varsigma-\mathbf{v}_s,V_\varsigma\right)\cdot J_\nu\left(\left(\mathbf{X_1},V_1\right),\ldots,\left(\mathbf{X}_\varsigma-\mathbf{v}_s,V_\varsigma\right),\ldots,\left(\mathbf{X}_\nu,V_\nu\right);t\right)$$
$$-a_s\left(\mathbf{X}_\varsigma,V_\varsigma\right)\cdot J_\nu\left(\left(\mathbf{X}_1,V_1\right),\ldots,\left(\mathbf{X}_\varsigma,V_\varsigma\right),\ldots,\left(\mathbf{X}_\nu,V_\nu\right);t\right)\Big]$$

$$F_G = -\sum_{\varsigma=1}^{\nu}\frac{\partial}{\partial V_\varsigma}\left[g\left(\mathbf{X}_\varsigma,V_\varsigma\right)\cdot J_\nu\left(\left(\mathbf{X}_1,V_1\right),\ldots,\left(\mathbf{X}_\varsigma,V_\varsigma\right),\ldots,\left(\mathbf{X}_\nu,V_\nu\right);t\right)\right]$$

$$F_D^{*} = 2\cdot\sum_{\varsigma=1}^{\nu-1}\sum_{\theta=\varsigma+1}^{\nu} a_d\left(\mathbf{X}_\varsigma+\mathbf{X}_\theta,V_\varsigma+V_\theta\right)\cdot h^{*}\left(\mathbf{X}_\varsigma,V_\varsigma\middle|\mathbf{X}_\varsigma+\mathbf{X}_\theta,V_\varsigma+V_\theta\right)\cdot$$
$$\cdot J_{\nu-1}\cdot\left(\left(\mathbf{X}_1,V_1\right),\ldots,\left(\mathbf{X}_\varsigma+\mathbf{X}_\theta,V_\varsigma+V_\theta\right),\ldots,\left(\mathbf{X}_{\theta-1},V_{\theta-1}\right),\left(\mathbf{X}_{\theta+1},V_{\theta+1}\right),\ldots,\left(\mathbf{X}_\nu,V_\nu\right);t\right)$$
$$-\sum_{\varsigma=1}^{\nu} a_d\left(\mathbf{X}_\varsigma,V_\varsigma\right)\cdot J_\nu\left(\left(\mathbf{X_1},V_1\right),\ldots,\left(\mathbf{X}_\varsigma,V_\varsigma\right),\ldots,\left(\mathbf{X}_\nu,V_\nu\right);t\right) \tag{22}$$

Simulation

Simulation of the CPME of Eq. 6 is performed with the algorithm described by Stamatakis and Zygourakis [2]. For the modified CPME Eq. 22, we used the same algorithm but without treating the chemical reaction as discrete stochastic events. Instead, we simulated continuous reaction kinetics by solving the ODEs of Eq. 15 between DNA duplication and division events.

Since the number of cells in a population increases exponentially with time, the computational cost becomes prohibitively large for long simulation times. Thus, the simulation often becomes intractable even before the cell population average reaches a stationary value. To overcome this limitation, we employed a constant number Monte Carlo scheme, in which a maximum number of observed cells (e.g. $N_{cellsmax}$ = 2000) is defined. If the population size surpasses the maximum after a division event, the algorithm removes randomly one cell from the population to restore

the population size to the maximum allowed number of cells [4-5]. This procedure results in a biased estimation of quantities such as the cell population average. However, the bias becomes negligible as the maximum number $N_{cellsmax}$ of observed cells increases.

Furthermore, for the CPME of Eq. 6 with stochastic reactions, the frequent simulation of the discrete reaction events makes the computational expense much higher than that of the modified CPME (22 for deterministic reaction rate expressions. Thus, one can only simulate a limited number of cells. In order to circumvent this problem, a multi-run scheme was employed. For a specific parameter set, a batch of several population simulations was initiated with the same initial condition (IC) but different seeds for the random number generator. The constant number MC technique was used when the population reached a number of cells $N_{cellsmax}$ = 500. Then, each population of the batch was sampled and, finally, all the samples obtained were used to calculate the number density function (NDF) of the batch. Thus, if the batch consisted of N_{batch} population simulations and at time t population i consisted of $N_{popul,i}$ cells, then the final NDF would be estimated as:

$$n(y,t)dy \triangleq \frac{1}{\sum_{i=1}^{N_{batch}} N_{popul,i}} \cdot \sum_{i=1}^{N_{batch}} \sum_{j=1}^{N_{popul,i}} \mathbf{1}_{\{y_{i,j} \in [y+dy)\}} \tag{23}$$

where y is the observable of interest (e.g. the total LacY concentration), $y_{i,j}$ is the value of the observable for cell j in population i, and $\mathbf{1}_{\{E\}}$ is an indicator taking value 1 if event E is true, 0 otherwise. Typical execution times ranged from 38 hrs of CPU time for the deterministic reaction dynamics to 360 hrs for the stochastic reactions.

SECTION S2: ASYMMETRIC (BIASED) PARTITIONING

As discussed in the previous section the partitioning of cell volume follows a symmetric beta distribution (equation 12).

$$\beta(V_d \mid V_m) = \frac{1}{V_m} \cdot \frac{\Gamma(2 \cdot q)}{(\Gamma(q))^2} \cdot \left(\frac{V_d}{V_m}\right)^{q-1} \cdot \left(1 - \frac{V_d}{V_m}\right)^{q-1} \tag{24}$$

It might be the case that one would want to model asymmetric (biased) partitioning, in which at every division event there is one daughter that consistently emerges larger after division. This situation can be modeled by a sum of asymmetric beta distributions:

$$\beta(V_d \mid V_m) = \frac{1}{2 \cdot V_m} \cdot \frac{\Gamma(a+b)}{\Gamma(a) \cdot \Gamma(b)} \cdot \left[\left(\frac{V_d}{V_m}\right)^{a-1} \cdot \left(1 - \frac{V_d}{V_m}\right)^{b-1} + \left(1 - \frac{V_d}{V_m}\right)^{a-1} \cdot \left(\frac{V_d}{V_m}\right)^{b-1}\right] \tag{25}$$

for a, b > 1. Then the expected volumes of the large and small daughters will respectively be:

$$\langle V_{d,large} \rangle = \frac{\max(a,b)}{a+b} \quad \text{and} \quad \langle V_{d,small} \rangle = \frac{\min(a,b)}{a+b} \tag{26}$$

Note that for $a = b = q$ equation (25) becomes identical to (24). Below, the two probability functions are plotted for demonstration purposes.

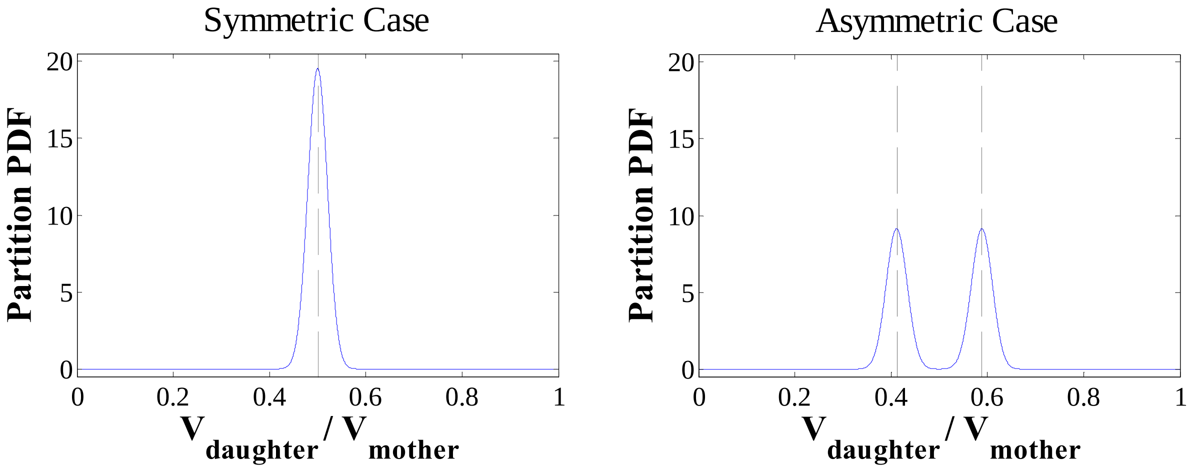

For the symmetric case $q = 300$. For the asymmetric case $a = 300$, $b = 0.7 \cdot a$.

SECTION S3: PARTITIONING IN THE POPULATION MODEL WITH DETERMINISTIC REACTION DYNAMICS

The population model with deterministic reaction dynamics can be thought as a limiting case of the one with stochastic reaction occurrence: the limit is taken for large numbers of molecules for all species. In this case, volume partitioning still follows a symmetric beta distribution (12); however, partitioning of the non-chromosomal DNA species will be governed by the limiting distribution of the binomial for a large number of trials. This limiting distribution is normal centered at

$$b_i\left(X_{d,i} \mid X_{m,i}, V_m, V_d\right) \doteq \frac{1}{\sqrt{2\cdot\pi\cdot\sigma^2_{X_{d,i}}}}\cdot\exp\left(-\frac{\left(X_{d,i}-\mu_{X_{d,i}}\right)^2}{2\cdot\sigma^2_{X_{d,i}}}\right) \qquad \text{for } i=1,\ldots,n \tag{27}$$

where:

$$\begin{aligned} \mu_{X_{d,i}} &= X_{m,i}\cdot\frac{V_d}{V_m} \\ \sigma^2_{X_{d,i}} &= X_{m,i}\cdot\frac{V_d}{V_m}\cdot\left(1-\frac{V_d}{V_m}\right) \end{aligned} \tag{28}$$

Similarly for the chromosomal DNA species:

$$c_i\left(X^{DNA,i}_{d,j} \mid \mathbf{X_m}, V_m, V_d\right) \doteq \frac{1}{\sqrt{2\cdot\pi\cdot\sigma^2_{X^{DNA,i}_{d,j}}}}\cdot\exp\left(-\frac{\left(X^{DNA,i}_{d,j}-\mu_{X^{DNA,i}_{d,j}}\right)^2}{2\cdot\sigma^2_{X_{d,i}}}\right)^{-1} \qquad \text{for } \begin{array}{l} i=1,\ldots,d \\ j=1,\ldots,s_i \end{array} \tag{29}$$

where:

$$\begin{aligned} \mu_{X^{DNA,i}_{d,j}} &= \frac{1}{2}\cdot X^{DNA,i}_{m,j} \\ \sigma^2_{X^{DNA,i}_{d,j}} &= \frac{1}{4}\cdot X^{DNA,i}_{m,j}\cdot\frac{\sum_{k=1}^{s_i} X^{DNA,i}_{m,k} - X^{DNA,i}_{m,j}}{\sum_{k=1}^{s_i} X^{DNA,i}_{m,k} - 1} \end{aligned} \tag{30}$$

For our purposes we are interested in the extreme case, where the numbers of molecules become so large that any stochastic fluctuations are suppressed (hence the deterministic reaction dynamics). In this limit, the distribution (27) collapses to a Dirac delta centered around $\mu_{X_{d,i}}$:

$$b_i\left(X_{d,i} \mid X_{m,i}, V_m, V_d\right) \doteq \delta\left(X_{d,i} - \frac{V_d}{V_m}\cdot X_{m,i}\right) \qquad \text{for } i = 1,\ldots,n \tag{31}$$

The above equation expresses that mother and daughter will have equal concentrations:

$$X_{d,i} - \frac{V_d}{V_m}\cdot X_{m,i} = 0 \Rightarrow \frac{X_{d,i}}{V_d} = \frac{X_{m,i}}{V_m} \tag{32}$$

Similarly for distribution (30):

$$c_i\left(\mathbf{X}_{\mathbf{d}}^{\mathbf{DNA,i}} \mid \mathbf{X}_{\mathbf{m}}, V_m, V_d\right) = \prod_{j=1}^{s_i}\delta\left(X_{d,j}^{DNA,i} - \frac{1}{2}\cdot X_{m,j}^{DNA,i}\right) \qquad \text{for } i = 1,\ldots,d \tag{33}$$

which shows that the random states of a DNA species are partitioned equally among the two daughters.

SECTION S4: STRUCTURED CONTINUUM MODEL

The mass balance equations for the structured continuum model are presented below. Variables in brackets denote population average intracellular species concentrations. Moreover, for this set of equations the mass balance of species R_2O was omitted since its concentration can be calculated by the conservation condition $[O]_T = [O] + [R_2O]$.

$$\frac{d[M_R]}{dt} = k_{sMR} - \lambda_{MR} \cdot [M_R] - g[M_R] \tag{34}$$

$$\frac{d[R]}{dt} = k_{sR} \cdot [M_R] - 2 \cdot k_{2R} \cdot [R]^2 + 2 \cdot k_{-2R} \cdot [R_2] - \lambda_R \cdot [R] - g \cdot [R] \tag{35}$$

$$\begin{aligned} \frac{d[R_2]}{dt} = {} & k_{2R} \cdot [R]^2 - k_{-2R} \cdot [R_2] - k_r \cdot [R_2] \cdot [O] + k_{-r} \cdot \left(\left\langle [O]_T \right\rangle - [O] \right) \\ & - k_{dr1} \cdot [R_2] \cdot [I]^2 + k_{-dr1} \cdot [I_2R_2] - \lambda_{R2} \cdot [R_2] - g \cdot [R_2] \end{aligned} \tag{36}$$

$$\begin{aligned} \frac{d[O]}{dt} = {} & -k_r \cdot [R_2] \cdot [O] + k_{-r} \cdot \left(\left\langle [O]_T \right\rangle - [O] \right) + k_{dr2} \cdot \left(\left\langle [O]_T \right\rangle - [O] \right) \cdot [I]^2 \\ & - k_{-dr2} \cdot [O] \cdot [I_2R_2] \end{aligned} \tag{37}$$

$$\begin{aligned} \frac{d[I]}{dt} = {} & -2 \cdot k_{dr1} \cdot [R_2] \cdot [I]^2 + 2 \cdot k_{-dr1} \cdot [I_2R_2] - 2 \cdot k_{dr2} \cdot \left(\left\langle [O]_T \right\rangle - [O] \right) \cdot [I]^2 \\ & + 2 \cdot k_{-dr2} \cdot [O] \cdot [I_2R_2] + k_{ft} \cdot [YI_{ex}] + h_t \cdot \left\langle \frac{A}{V} \right\rangle \cdot \left([I_{ex}] - [I] \right) \\ & + 2 \cdot \lambda_{I2R2} \cdot [I_2R_2] + \lambda_{YI_{ex}} \cdot [YI_{ex}] - g \cdot [I] \end{aligned} \tag{38}$$

$$\begin{aligned} \frac{d[I_2R_2]}{dt} = {} & k_{dr1} \cdot [R_2] \cdot [I]^2 - k_{-dr1} \cdot [I_2R_2] + k_{dr2} \cdot \left(\left\langle [O]_T \right\rangle - [O] \right) \cdot [I]^2 \\ & - k_{-dr2} \cdot [O] \cdot [I_2R_2] - \lambda_{I2R2} \cdot [I_2R_2] - g \cdot [I_2R_2] \end{aligned} \tag{39}$$

$$\frac{d[M_Y]}{dt} = k_{s0MY} \cdot \left(\left\langle [O]_T \right\rangle - [O] \right) + k_{s1MY} \cdot [O] - \lambda_{MY} \cdot [M_Y] - g \cdot [M_Y] \tag{40}$$

$$\frac{d[Y]}{dt} = k_{sY} \cdot [M_Y] + \left(k_{ft} + k_{-p} \right) \cdot [YI_{ex}] - k_p \cdot [Y] \cdot [I_{ex}] - \lambda_Y \cdot [Y] - g \cdot [Y] \tag{41}$$

$$\frac{d[YI_{ex}]}{dt} = -\left(k_{ft} + k_{-p} \right) \cdot [YI_{ex}] + k_p \cdot [Y] \cdot [I_{ex}] - \lambda_{YIex} \cdot [YI_{ex}] - g \cdot [YI_{ex}] \tag{42}$$

REFERENCES

1. Ramkrishna D: *Population Balances: Theory and Applications to Particulate Systems in Engineering.* San Diego, CA: Academic Press; 2000.
2. Stamatakis M, Zygourakis K: **A Mathematical and Computational Approach for Integrating the Major Sources of Cell Population Heterogeneity.** *Journal of Theoretical Biology* 2010, **266:**41-61.
3. Cooper S: **What is the bacterial growth law during the division cycle?** *J Bacteriol* 1988, **170:**5001-5005.
4. Lee K, Matsoukas T: **Simultaneous coagulation and break-up using constant-N Monte Carlo.** *Powder Technol* 2000, **110:**82-89.
5. Smith M, Matsoukas T: **Constant-number Monte Carlo simulation of population balances.** *Chemical Engineering Science* 1998, **53:**1777-1786.
